# Bis[1-(2-eth­oxy­phen­yl)-3-(4-nitro­phen­yl)triazenido]mercury(II)

**DOI:** 10.1107/S1600536810031326

**Published:** 2010-08-11

**Authors:** Mohammad Kazem Rofouei, Ehsan Fereyduni, Jafar Attar Gharamaleki, Giuseppe Bruno, Hadi Amiri Rudbari

**Affiliations:** a Faculty of Chemistry, Tarbiat Moallem University, Tehran, Iran; b Young Researchers Club, Islamic Azad University, North Tehran Branch, Tehran, Iran; c Dipartimento di Chimica Inorganica, Vill. S. Agata, Salita Sperone 31, Universita di Messina 98166 Messina, Italy

## Abstract

In the title compound, [Hg(C_14_H_13_N_4_O_3_)_2_], the central Hg atom (site symmetry 2) is six-coordinated by two tridentate 1-(2-eth­oxy­phen­yl)-3-(4-nitro­phen­yl)triazenide ligands through two N and one O atoms. The mononuclear complex mol­ecules are connected into two parallel chains by inter­molecular C—H⋯O hydrogen-bonding inter­actions. These chains are connected to each other by face-to-edge C—H⋯π inter­actions between the CH of the ethoxy groups and the aromatic rings, resulting in a two-dimensional architecture in the *ac* plane.

## Related literature

For related structures, see: Melardi *et al.* (2007 ▶, 2009 ▶); Rofouei *et al.* (2009 ▶). For a similar complex with the same ligand, see: Melardi *et al.* (2010 ▶).
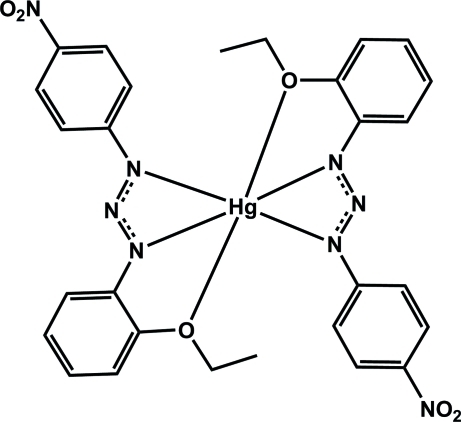


## Experimental

### 

#### Crystal data

[Hg(C_14_H_13_N_4_O_3_)_2_]
*M* *_r_* = 771.1
Orthorhombic, 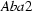

*a* = 15.4637 (3) Å
*b* = 18.6594 (4) Å
*c* = 9.8008 (2) Å
*V* = 2827.96 (10) Å^3^
*Z* = 4
Mo *K*α radiation
μ = 5.50 mm^−1^
*T* = 296 K
0.50 × 0.45 × 0.20 mm

#### Data collection

Bruker APEXII CCD diffractometer
Absorption correction: integration (*SADABS*; Bruker, 2005 ▶) *T*_min_ = 0.129, *T*_max_ = 0.280
51881 measured reflections
3358 independent reflections
2629 reflections with *I* > 2σ(*I*)
*R*_int_ = 0.122

#### Refinement

*R*[*F* ^2^ > 2σ(*F* ^2^)] = 0.038
*wR*(*F* ^2^) = 0.110
*S* = 1.15
3358 reflections
196 parameters
1 restraint
H-atom parameters constrained
Δρ_max_ = 2.28 e Å^−3^
Δρ_min_ = −2.52 e Å^−3^
Absolute structure: Flack (1983 ▶), 1575 Friedel pairs
Flack parameter: −0.08 (2)

### 

Data collection: *APEX2* (Bruker, 2005 ▶); cell refinement: *SAINT-Plus* (Bruker, 2001 ▶); data reduction: *SAINT-Plus*; program(s) used to solve structure: *SHELXS97* (Sheldrick, 2008 ▶); program(s) used to refine structure: *SHELXL97* (Sheldrick, 2008 ▶); molecular graphics: *SHELXTL* (Sheldrick, 2008 ▶); software used to prepare material for publication: *SHELXTL*.

## Supplementary Material

Crystal structure: contains datablocks I, global. DOI: 10.1107/S1600536810031326/pv2312sup1.cif

Structure factors: contains datablocks I. DOI: 10.1107/S1600536810031326/pv2312Isup2.hkl

Additional supplementary materials:  crystallographic information; 3D view; checkCIF report

## Figures and Tables

**Table 1:** Hydrogen-bond geometry (Å, °) *Cg*1 is the centroid of the C1–C6 ring.

*D*—H⋯*A*	*D*—H	H⋯*A*	*D*⋯*A*	*D*—H⋯*A*
C8—H8⋯O1^i^	0.93	2.52	3.446 (9)	177
C13—H13*A*⋯*Cg*1^ii^	0.97	2.86	3.764 (8)	155

Symmetry codes: (i) 
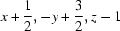
; (ii) 
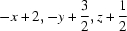
.

**Fig. 1.**
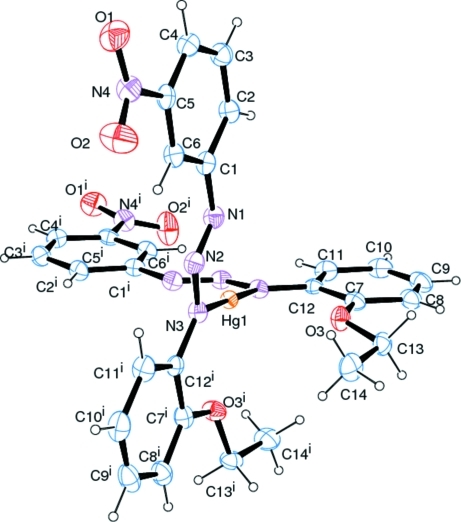
Molecular structure of the title compound, with ellipsoids drawn at 50% probability level.

**Fig. 2.**
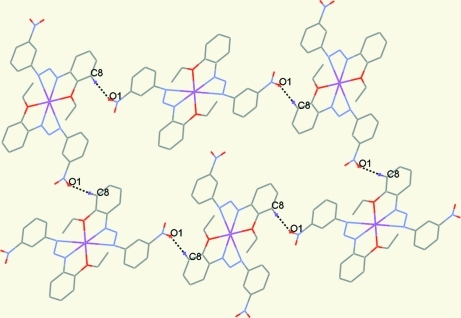
C8—H8···O1 Non-classical hydrogen bonds between Hg(C14H13N4O3)2 moieties.

**Fig. 3.**
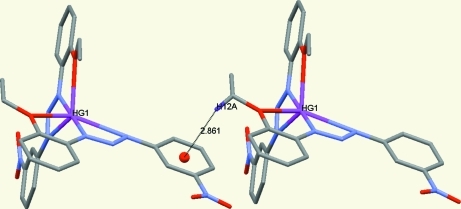
C—H···π stacking interactions between two Hg(C14H13N4O3)2 moieties.

**Fig. 4.**
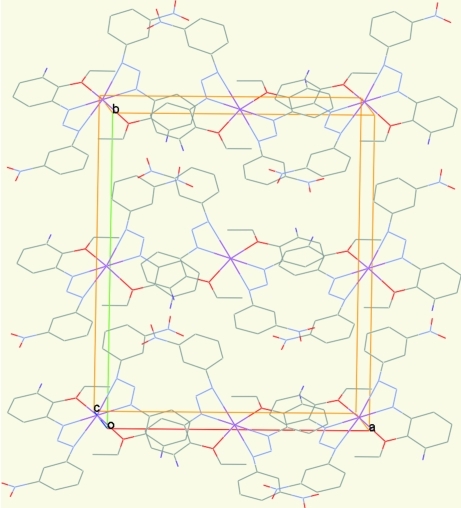
The unit cell packing diagram of the title compound along the c axis. Hydrogen atoms not involving in the hydrogen bonds are omitted for clarity.

## supplementary crystallographic information

### Comment

The study of transition-metal complexes containing 1,3-diaryltriazene ligands has greatly increased in the past few years because of the versatility of their coordination forms, yielding a variety of coordination compounds with large structural diversity. The crystal structures of a few complexes related to the title compound have been reported recently from our laboratory (Melardi *et al.*, 2007, 2009, 2010; Rofouei *et al.*, 2009).

In the title complex (Fig. 1), two [1-(2-ethoxyphenyl)3-(4-nitrophenyl)triazenido] ligands are coordinated to the central atom Hg(II), each through two N atoms [Hg1—N1 = 2.7873 (1) Å and Hg1—N3 = 2.0836 (1) Å] and one O atom [Hg1—O1 = 2.6562 (1) Å]. The Hg1—N1 is significantly longer than the Hg1—N3 bond. In the lattice of the title compound, the monomeric Hg(C_14_H_13_N_4_O_3_)_2_ moieties are linked into chains through non-classical C8—H8···O1 hydrogen bonds, as well as C—H···π stacking interactions. Moreover, the mono-nuclear complexe molceules are connected to form two parallel chains by distinct intermolecular non-classical C—H···O hydrogen bonds. Consequently, 1-D chains are connected with one another by C—H···π stacking interactions, resulting in a 2-D architecture. These C—H···π edge-to-face interactions are present between CH group of ethoxy with aromatic rings with H···π distance of 2.86 Å for C13—H12A···*Cg*1 (2 - *x*,3/2 - *y*, *z-*1/2) [*Cg*1= C1—C6 (Tab. 1, Figs. 2 and 3). Also, the sum of the weak non-covalent interactions seems to play an important role in the crystal packing and the formation of a formed framework. The unit cell packing diagram of the title compound is shown in Fig. 4.

### Experimental

The title complex was prepared by dissolving [1-(2-ethoxyphenyl)3- (4-nitrophenyl)]triazene (0.58 g, 2 mmol) in 20 ml anhydrous methanol. A solution of mercury acetate (0.32 g, 1 mmol) in 20 ml anhydrous methanol was added to the ligand solution. After 1 h, a red-orange solid was readily precipitated out. After two weeks beautiful red-orange and air-stable crystals of the title complex were obtained by slow evaporation of the solvent.

### Refinement

An absolute structure was established using Flack (1983) method. The H-atoms were placed in calculated positions with C—H = 0.93, 0.96 and 0.97 Å for aryl, methyl and methylene type H-atoms, respectively, and included in the refinement in riding mode with isotropic displacement parameters *U*_iso_(H) = 1.5 and 1.2 × *U*_eq_(C) for the CH_3_ and other groups, respectively. The final difference map showed electron density within 1.0 Å from Hg1 atom and may be attributed to absorption effects.

### Crystal data

**Table:**

[Hg(C_14_H_13_N_4_O_3_)_2_]	*F*(000) = 1512
*M**_r_* = 771.1	*D*_x_ = 1.811 Mg m^−^^3^*D*_m_ = 1.8 Mg m^−^^3^*D*_m_ measured by not measured
Orthorhombic, *A**b**a*2	Mo *K*α radiation, λ = 0.71073 Å
Hall symbol: A 2 -2ac	Cell parameters from 9751 reflections
*a* = 15.4637 (3) Å	θ = 2.6–27.8°
*b* = 18.6594 (4) Å	µ = 5.50 mm^−^^1^
*c* = 9.8008 (2) Å	*T* = 296 K
*V* = 2827.96 (10) Å^3^	Irregular, red
*Z* = 4	0.50 × 0.45 × 0.20 mm

### Data collection

**Table:**

Bruker APEXII CCD diffractometer	3358 independent reflections
Radiation source: fine-focus sealed tube	2629 reflections with *I* > 2σ(*I*)
graphite	*R*_int_ = 0.122
φ and ω scans	θ_max_ = 27.9°, θ_min_ = 2.6°
Absorption correction: integration (*SADABS*; Bruker, 2005)	*h* = −20→20
*T*_min_ = 0.129, *T*_max_ = 0.280	*k* = −24→24
51881 measured reflections	*l* = −12→12

### Refinement

**Table:**

Refinement on *F*^2^	Hydrogen site location: inferred from neighbouring sites
Least-squares matrix: full	H-atom parameters constrained
*R*[*F*^2^ > 2σ(*F*^2^)] = 0.038	*w* = 1/[σ^2^(*F*_o_^2^) + (0.0655*P*)^2^ + 3.2492*P*] where *P* = (*F*_o_^2^ + 2*F*_c_^2^)/3
*wR*(*F*^2^) = 0.110	(Δ/σ)_max_ < 0.001
*S* = 1.15	Δρ_max_ = 2.28 e Å^−^^3^
3358 reflections	Δρ_min_ = −2.52 e Å^−^^3^
196 parameters	Extinction correction: *SHELXL97* (Sheldrick, 2008), Fc^*^=kFc[1+0.001xFc^2^λ^3^/sin(2θ)]^-1/4^
1 restraint	Extinction coefficient: 0.00070 (17)
Primary atom site location: structure-invariant direct methods	Absolute structure: Flack (1983), 1575 Friedel pairs
Secondary atom site location: difference Fourier map	Flack parameter: −0.08 (2)

### Special details

**Table:**

Geometry. All s.u.'s (except the s.u. in the dihedral angle between two l.s. planes) are estimated using the full covariance matrix. The cell s.u.'s are taken into account individually in the estimation of s.u.'s in distances, angles and torsion angles; correlations between s.u.'s in cell parameters are only used when they are defined by crystal symmetry. An approximate (isotropic) treatment of cell s.u.'s is used for estimating s.u.'s involving l.s. planes.
Refinement. Refinement of *F*^2^ against ALL reflections. The weighted *R*-factor *wR* and goodness of fit *S* are based on *F*^2^, conventional *R*-factors *R* are based on *F*, with *F* set to zero for negative *F*^2^. The threshold expression of *F*^2^ > σ(*F*^2^) is used only for calculating *R*-factors(gt) *etc*. and is not relevant to the choice of reflections for refinement. *R*-factors based on *F*^2^ are statistically about twice as large as those based on *F*, and *R*- factors based on ALL data will be even larger.

### Fractional atomic coordinates and isotropic or equivalent isotropic displacement parameters (Å^2^)

**Table:**

	*x*	*y*	*z*	*U*_iso_*/*U*_eq_	
Hg1	1.0000	1.0000	0.7679 (3)	0.03974 (13)	
C7	1.1773 (4)	0.9517 (3)	0.5961 (6)	0.0546 (14)	
C2	0.9670 (4)	0.8059 (3)	1.0850 (7)	0.0545 (14)	
H2	1.0236	0.8178	1.0624	0.065*	
O1	0.7000 (3)	0.7116 (3)	1.2795 (6)	0.0925 (17)	
O3	1.0915 (3)	0.9353 (2)	0.5744 (5)	0.0615 (11)	
N1	0.9199 (3)	0.8912 (2)	0.9162 (5)	0.0554 (11)	
C3	0.9533 (5)	0.7573 (3)	1.1860 (7)	0.0627 (16)	
H3	0.9999	0.7371	1.2319	0.075*	
C14	0.9689 (8)	0.8927 (5)	0.4560 (11)	0.080 (3)	
H14A	0.9497	0.8639	0.3806	0.120*	
H14B	0.9488	0.9410	0.4444	0.120*	
H14C	0.9462	0.8734	0.5394	0.120*	
C8	1.2429 (5)	0.9181 (3)	0.5204 (8)	0.0659 (15)	
H8	1.2297	0.8841	0.4543	0.079*	
N3	0.8716 (3)	0.9660 (2)	0.7669 (6)	0.0492 (9)	
N2	0.8539 (3)	0.9140 (3)	0.8513 (5)	0.0536 (11)	
C1	0.8989 (3)	0.8390 (3)	1.0133 (6)	0.0505 (12)	
C4	0.8678 (5)	0.7375 (3)	1.2210 (7)	0.0586 (15)	
H4	0.8568	0.7048	1.2905	0.070*	
C5	0.8026 (5)	0.7683 (4)	1.1489 (8)	0.0528 (16)	
N4	0.7125 (5)	0.7478 (4)	1.1825 (7)	0.0661 (18)	
O2	0.6572 (3)	0.7650 (4)	1.1014 (9)	0.116 (3)	
C6	0.8148 (3)	0.8168 (3)	1.0484 (7)	0.0526 (13)	
H6	0.7675	0.8357	1.0022	0.063*	
C13	1.0658 (5)	0.8922 (4)	0.4614 (8)	0.0605 (18)	
H13A	1.0869	0.8436	0.4728	0.073*	
H13B	1.0895	0.9115	0.3774	0.073*	
C9	1.3285 (5)	0.9371 (4)	0.5470 (9)	0.080 (2)	
H9	1.3728	0.9154	0.4979	0.096*	
C12	1.1960 (5)	1.0019 (2)	0.6943 (9)	0.0420 (15)	
C11	1.2818 (5)	1.0216 (5)	0.7181 (9)	0.0652 (17)	
H11	1.2950	1.0569	0.7818	0.078*	
C10	1.3485 (7)	0.9873 (5)	0.6443 (13)	0.075 (3)	
H10	1.4060	0.9988	0.6616	0.090*	

### Atomic displacement parameters (Å^2^)

**Table:**

	*U*^11^	*U*^22^	*U*^33^	*U*^12^	*U*^13^	*U*^23^
Hg1	0.03350 (17)	0.04451 (17)	0.04121 (18)	−0.00398 (7)	0.000	0.000
C7	0.047 (3)	0.053 (3)	0.064 (4)	−0.002 (2)	0.000 (3)	0.013 (3)
C2	0.033 (3)	0.063 (3)	0.068 (4)	−0.002 (2)	−0.001 (3)	−0.005 (3)
O1	0.080 (3)	0.101 (4)	0.097 (4)	−0.019 (3)	0.029 (3)	0.012 (3)
O3	0.045 (2)	0.068 (2)	0.071 (3)	−0.0021 (18)	0.0040 (18)	−0.016 (2)
N1	0.047 (2)	0.059 (3)	0.061 (3)	−0.006 (2)	0.009 (2)	0.000 (3)
C3	0.052 (4)	0.069 (4)	0.067 (4)	0.003 (3)	0.000 (3)	−0.001 (3)
C14	0.081 (6)	0.073 (5)	0.086 (6)	−0.008 (5)	−0.014 (6)	−0.029 (4)
C8	0.061 (4)	0.065 (3)	0.071 (3)	0.008 (3)	0.009 (3)	0.017 (4)
N3	0.037 (2)	0.053 (2)	0.058 (3)	−0.0038 (17)	0.002 (2)	−0.006 (3)
N2	0.048 (3)	0.057 (3)	0.056 (3)	−0.008 (2)	0.008 (2)	−0.004 (2)
C1	0.042 (3)	0.053 (3)	0.057 (3)	−0.010 (2)	0.007 (2)	−0.009 (3)
C4	0.061 (4)	0.058 (3)	0.057 (4)	−0.009 (3)	0.005 (3)	−0.002 (3)
C5	0.042 (3)	0.054 (3)	0.062 (4)	−0.004 (2)	0.013 (3)	−0.012 (3)
N4	0.047 (4)	0.070 (4)	0.081 (5)	−0.011 (2)	0.020 (3)	0.012 (3)
O2	0.040 (3)	0.148 (5)	0.159 (6)	−0.005 (3)	0.012 (4)	0.066 (5)
C6	0.035 (2)	0.056 (3)	0.067 (4)	−0.006 (2)	0.007 (2)	−0.004 (3)
C13	0.075 (5)	0.044 (3)	0.062 (4)	−0.001 (3)	0.009 (4)	−0.009 (3)
C9	0.061 (4)	0.082 (5)	0.096 (6)	0.020 (4)	0.023 (4)	0.036 (5)
C12	0.039 (4)	0.046 (3)	0.041 (4)	−0.0032 (18)	0.000 (3)	0.0117 (19)
C11	0.043 (4)	0.081 (4)	0.071 (5)	−0.008 (4)	0.000 (3)	0.012 (4)
C10	0.040 (4)	0.103 (6)	0.082 (7)	−0.005 (4)	0.002 (4)	0.032 (5)

### Geometric parameters (Å, °)

**Table:**

Hg1—N3^i^	2.084 (4)	C8—C9	1.394 (11)
Hg1—N3	2.084 (4)	C8—H8	0.9300
Hg1—O3	2.656 (5)	N3—N2	1.304 (6)
Hg1—O3^i^	2.656 (5)	N3—C12^i^	1.400 (9)
C7—C12	1.374 (9)	C1—C6	1.407 (6)
C7—O3	1.378 (7)	C4—C5	1.359 (11)
C7—C8	1.406 (9)	C4—H4	0.9300
C2—C3	1.359 (9)	C5—C6	1.351 (10)
C2—C1	1.409 (8)	C5—N4	1.481 (10)
C2—H2	0.9300	N4—O2	1.211 (10)
O1—N4	1.182 (9)	C6—H6	0.9300
O3—C13	1.425 (8)	C13—H13A	0.9700
N1—N2	1.275 (6)	C13—H13B	0.9700
N1—C1	1.400 (7)	C9—C10	1.372 (14)
C3—C4	1.414 (9)	C9—H9	0.9300
C3—H3	0.9300	C12—C11	1.396 (11)
C14—C13	1.500 (13)	C12—N3^i^	1.400 (9)
C14—H14A	0.9600	C11—C10	1.412 (15)
C14—H14B	0.9600	C11—H11	0.9300
C14—H14C	0.9600	C10—H10	0.9300
			
N3^i^—Hg1—N3	179.4 (4)	N1—C1—C2	118.1 (5)
N3^i^—Hg1—O3	68.12 (18)	C6—C1—C2	116.1 (6)
N3—Hg1—O3	111.46 (18)	C5—C4—C3	117.2 (6)
N3^i^—Hg1—O3^i^	111.46 (18)	C5—C4—H4	121.4
N3—Hg1—O3^i^	68.12 (18)	C3—C4—H4	121.4
O3—Hg1—O3^i^	88.9 (2)	C6—C5—C4	123.9 (6)
C12—C7—O3	117.6 (6)	C6—C5—N4	117.8 (7)
C12—C7—C8	121.5 (6)	C4—C5—N4	118.2 (7)
O3—C7—C8	121.0 (6)	O1—N4—O2	124.3 (7)
C3—C2—C1	122.6 (6)	O1—N4—C5	118.7 (7)
C3—C2—H2	118.7	O2—N4—C5	116.8 (6)
C1—C2—H2	118.7	C5—C6—C1	120.3 (6)
C7—O3—C13	120.9 (5)	C5—C6—H6	119.8
C7—O3—Hg1	107.5 (4)	C1—C6—H6	119.8
C13—O3—Hg1	131.5 (4)	O3—C13—C14	107.6 (5)
N2—N1—C1	112.7 (4)	O3—C13—H13A	110.2
C2—C3—C4	119.8 (6)	C14—C13—H13A	110.2
C2—C3—H3	120.1	O3—C13—H13B	110.2
C4—C3—H3	120.1	C14—C13—H13B	110.2
C13—C14—H14A	109.5	H13A—C13—H13B	108.5
C13—C14—H14B	109.5	C10—C9—C8	121.2 (8)
H14A—C14—H14B	109.5	C10—C9—H9	119.4
C13—C14—H14C	109.5	C8—C9—H9	119.4
H14A—C14—H14C	109.5	C7—C12—C11	119.8 (8)
H14B—C14—H14C	109.5	C7—C12—N3^i^	119.3 (6)
C9—C8—C7	118.2 (7)	C11—C12—N3^i^	120.9 (7)
C9—C8—H8	120.9	C12—C11—C10	119.2 (9)
C7—C8—H8	120.9	C12—C11—H11	120.4
N2—N3—C12^i^	118.9 (5)	C10—C11—H11	120.4
N2—N3—Hg1	115.1 (4)	C9—C10—C11	120.1 (9)
C12^i^—N3—Hg1	125.8 (4)	C9—C10—H10	120.0
N1—N2—N3	113.4 (4)	C11—C10—H10	120.0
N1—C1—C6	125.8 (5)		
			
C12—C7—O3—C13	170.6 (6)	C3—C2—C1—C6	−2.4 (9)
C8—C7—O3—C13	−9.5 (8)	C2—C3—C4—C5	0.8 (10)
C12—C7—O3—Hg1	−8.0 (6)	C3—C4—C5—C6	−1.0 (11)
C8—C7—O3—Hg1	172.0 (4)	C3—C4—C5—N4	179.2 (6)
N3^i^—Hg1—O3—C7	10.0 (3)	C6—C5—N4—O1	−171.7 (7)
N3—Hg1—O3—C7	−170.4 (3)	C4—C5—N4—O1	8.0 (10)
O3^i^—Hg1—O3—C7	123.7 (4)	C6—C5—N4—O2	13.8 (11)
N3^i^—Hg1—O3—C13	−168.4 (6)	C4—C5—N4—O2	−166.5 (7)
N3—Hg1—O3—C13	11.3 (6)	C4—C5—C6—C1	−0.5 (10)
O3^i^—Hg1—O3—C13	−54.6 (5)	N4—C5—C6—C1	179.2 (6)
C1—C2—C3—C4	0.9 (9)	N1—C1—C6—C5	−176.8 (6)
C12—C7—C8—C9	−0.2 (9)	C2—C1—C6—C5	2.2 (8)
O3—C7—C8—C9	179.8 (5)	C7—O3—C13—C14	−172.6 (6)
O3—Hg1—N3—N2	94.9 (4)	Hg1—O3—C13—C14	5.6 (8)
O3^i^—Hg1—N3—N2	174.5 (4)	C7—C8—C9—C10	−0.1 (10)
O3—Hg1—N3—C12^i^	−91.2 (5)	O3—C7—C12—C11	−178.5 (6)
O3^i^—Hg1—N3—C12^i^	−11.6 (5)	C8—C7—C12—C11	1.5 (9)
C1—N1—N2—N3	175.7 (4)	O3—C7—C12—N3^i^	−0.1 (8)
C12^i^—N3—N2—N1	−177.0 (5)	C8—C7—C12—N3^i^	179.9 (6)
Hg1—N3—N2—N1	−2.6 (6)	C7—C12—C11—C10	−2.5 (11)
N2—N1—C1—C6	−5.5 (7)	N3^i^—C12—C11—C10	179.1 (7)
N2—N1—C1—C2	175.6 (5)	C8—C9—C10—C11	−1.0 (12)
C3—C2—C1—N1	176.6 (5)	C12—C11—C10—C9	2.3 (13)

Symmetry codes: (i) −*x*+2, −*y*+2, *z*.

### Hydrogen-bond geometry (Å, °)

**Table:**

*D*—H···*A*	*D*—H	H···*A*	*D*···*A*	*D*—H···*A*
C8—H8···O1^ii^	0.93	2.52	3.446 (9)	177
C13—H13A···Cg1^iii^	0.97	2.86	3.764 (8)	155

Symmetry codes: (ii) *x*+1/2, −*y*+3/2, *z*−1; (iii) −*x*+2, −*y*+3/2, *z*+1/2.
